# Polymerase Chain Reaction Assay and Bacterial Meningitis Surveillance in Remote Areas, Niger

**DOI:** 10.3201/eid0911.030462

**Published:** 2003-11

**Authors:** Fati Sidikou, Saacou Djibo, Muhamed Kheir Taha, Jean Michel Alonso, Ali Djibo, Kiari Kaka Kairo, Suzanne Chanteau, Pascal Boisier

**Affiliations:** * Centre de Recherches Médicales et Sanitaires, Niamey, Niger; † Institut Pasteur, Paris, France; ‡ Ministère de la Santé, Niamey, Niger

## Abstract

To compensate for the lack of laboratories in remote areas, the national reference laboratory for meningitis in Niger used polymerase chain reaction (PCR) to enhance the surveillance of meningitis caused by *Neisseria meningitidis*, *Streptococcus pneumoniae,* and *Haemophilus influenzae*. PCR effectively documented the wide geographic spread of *N. meningitidis* serogroup W135.

Niger is a sub-Saharan country located in the center of the African “meningitis belt,” which includes all or parts of 18 sub-Saharan countries (*1*). The population of approximately 11 million is mainly concentrated in the southern part of the territory; 84% of the population lives in rural areas. Every year, during the dry, warm, and windy season, between January and May, localized epidemics of meningococcal meningitis occur in several districts, whereas major epidemics, affecting the entire territory, break out at regular intervals of every few years (*2*,*3*). Previously, all important epidemics have been caused by *Neisseria meningitidis* serogroup A, and occasionally, small outbreaks have been caused by serogroup X isolates (*4*). However, sporadic cases of meningitis caused by serogroup *N. meningitidis* W135 have been laboratory-confirmed in Niger since the beginning of the 1980s (*5*). Strains of serogroup W135 were found as often as strains of serogroup A at the end of the epidemic of 2001 (*6*). In 2002 and again in 2003, Burkina Faso experienced the first large epidemics caused by *N. meningitidis* serogroup W135 in Africa (*7*,*8*). This event is a cause of concern for public health authorities of neighboring countries, including Niger, and for the World Health Organization because current affordable meningococcal vaccines available in large amounts target only *N. meningitidis* serogroups A and C and do not protect against serogroup W135. On the other hand, vaccines including the *N. meningitidis* serogroup W135 valency are not only expensive but also rare, and this situation will likely continue for the next 3 to 4 years. Enhanced microbiologic surveillance is needed to quickly identify the serogroup of *N. meningitidis* involved so that the appropriate vaccine for emergency mass vaccination can be selected (*9*).

In Niger, few laboratories are able to perform etiologic diagnosis of bacterial meningitis, although the country is large (1,267 km^2^) and the population is scattered. Until 2002, microbiologic surveillance existed but was inadequate because it focused almost entirely on the capital city. The Centre de Recherches Médicales et Sanitaires (CERMES) in Niamey became, in 2002, a national biomedical research center under the authority of the Ministry of Health (MOH) and the national reference center for meningitis in Niger. The polymerase chain reaction (PCR) method for the diagnosis of acute bacterial meningitis was transferred from the Institut Pasteur (Paris, France) for research purposes in October 2002. Therefore, CERMES decided to include the PCR assay in the national framework of the routine surveillance system of the MOH in November 2002 to be ready when the next meningitis season began in January. The MOH has asked physicians and nurses to systematically keep frozen (or at least refrigerated in small health facilities) every sample of cerebrospinal fluid (CSF) collected from patients with suspected cases of acute meningitis. Most often, in remote healthcare centers and in the absence of laboratories, the clarity of the CSF was assessed by macroscopic examination only, so the entire volume of CSF that was previously discarded was kept for PCR analysis. In the few district hospitals that have appropriate laboratory facilities, the remaining CSF samples were stored after the latex agglutination assay or bacteriologic tests had been performed. After laboratory personnel had been informed of the purpose of the surveillance, sterile tubes and epidemiologic forms were set up in 14 health districts within a radius of approximately 250 km of Niamey. The designated area represented approximately 35% of the whole population. Afterwards, the CSF samples were regularly collected by a CERMES vehicle, according to a precise timetable. Later, tubes and forms were also set up in all health regions of Niger, and the healthcare centers located beyond the limit of 250 km used any suitable opportunities to convey the CSF samples to CERMES. Thus, the microbiologic surveillance included >50% of the population. The implementation of this strategy and the use of the results were carried out in close collaboration with the national surveillance system.

CSF specimens were tested by PCR (amplification for 35 cycles) for the three main causative agents of acute bacterial meningitis in Niger: *N. meningitidis* (*10*), *Streptococcus pneumoniae* (*11*), and *Haemophilus influenzae* (*12*). A second PCR was performed on specimens positive for *N. meningitidis* to identify serogroups A, B, C, and Y/W135, and finally, serogroups Y/W135 were differentiated from both Y and W135 alone by a confirmative PCR (*10*). In the same manner, specimens positive for *H. influenzae* were further tested for type b DNA.

From November 2002 to May 2003, which included the entire season of transmission, 1,651 CSF specimens collected within the national surveillance system were processed by using PCR; 1,239 (75%) specimens came from outside the capital, Niamey. Until mid-2002, most of these specimens would have been lost for microbiologic surveillance. A total of 778 specimens (47.1%) were positive: 661 for *N. meningitidis* (85%), 83 for *S. pneumoniae* (10.1%), and 34 for *H. influenzae* (4.4%). The results of CSF examinations, according to the month and region, are presented in Figures 1 and 2. Surveillance highlighted that the meningococcal serogroup *N. meningitidis* W135 accounted for 8.5% of all *N. meningitidis* and that W135 was found in most of the regions in Niger, although it did not cause epidemics. *N. meningitidis* serogroup W135 was rare or absent in regions where *N. meningitidis* serogroup A epidemics occurred (three serogroup W135 isolates among 368 *N. meningitidis* isolates in Zinder and no W135 among 79 *N. meningitidis* isolates in Maradi). With 284 specimens from Niamey, which had undergone both PCR and bacteriologic testing, we compared the results from the two methods. The results of both tests were in agreement for 231 specimens (81.3%); results for 182 of those specimens were negative. PCR found 25 positive samples for which bacteriologic tests were negative, and 8 more positive specimens on which bacteriologic testing could not be carried out because of contamination. Conversely, bacteriologic testing provided a diagnosis for six PCR-negative samples. However, the region of Niamey presented the lowest overall rate of confirmation of suspected bacterial meningitis so this comparison is less conclusive. The two diagnostic methods were also applied to 102 specimens sent on trans-isolate medium. As is frequently observed, a contamination problem occurred, and 32 samples (31.3%) were unsuitable for bacteriologic testing, but PCR identified a causative agent in 20 of these 32 cases. Of the remaining samples, for 46 (65.7%), bacteriologic testing and PCR results were concordant, whereas 19 samples negative by bacteriologic testing, tested positive by PCR. The opposite was observed for two specimens.

**Figure 1:**
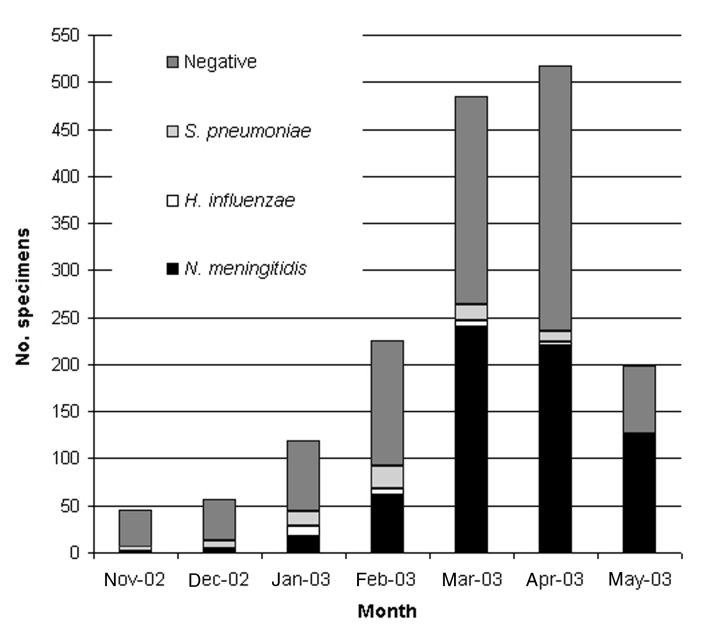
Results of polymerase chain reaction assay on cerebrospinal fluid specimens, November 2002–May 2003. *S, Streptococcus; H, Haemophilus; N, Neisseria*.

**Figure 2:**
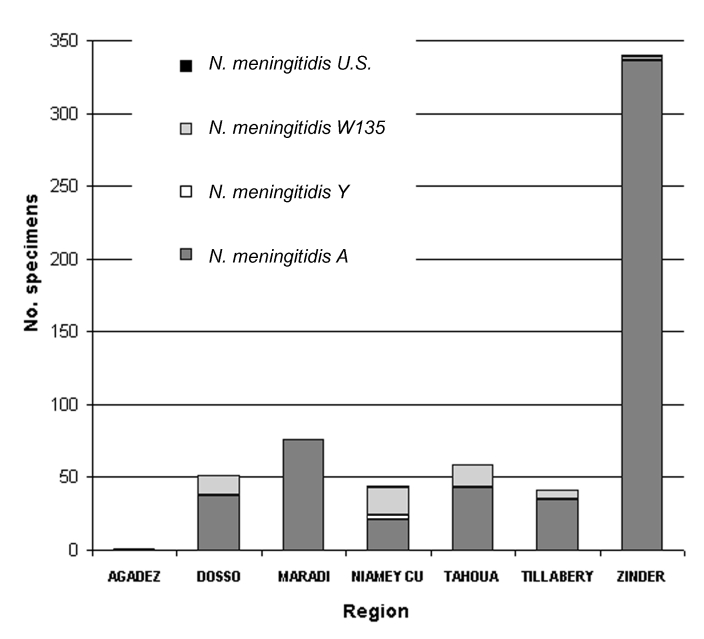
Distribution of serogroups of *Neisseria meningitidis* according to region, November 2002–May 2003. (*N. meningitidis* U.S. = unpredicted serogroup, i.e., not A, not B, not C, not Y and not W135.)

The overall proportion of specimens that tested positive for one of the three species of bacteria varied according to the period. From November to February, before the meningitis epidemic season, the positivity rate was low in all the regions. In March and April, during the epidemic season, the positivity rate remained low in the districts not undergoing epidemics, although the positivity rate reached 60.9% in the Zinder region, where epidemics due to *N. meningitidis* serogroup A occurred. Because of the uncertainty of the cold chain in remote areas, some specimens may have been stored without taking into account the temperature conditions, which might have affected the sensitivity of the PCR testing. An analysis of confirmation rates must also take into account that CSF samples were tested whether they were cloudy or purulent or not and that the currently used PCR focuses on three species only, although many other causes of meningitis exist. The proportion of negative results may also indicate that the national health services keep a vigilant watch over meningitis and that the diagnosis is widely used, perhaps excessively. The symptoms are not fully specific, and the predictive value of the clinical picture for meningococcal meningitis increases substantially during an epidemic, as shown by the results from Zinder.

In contrast to the culture method, which requires that laboratories receive live bacteria (quite restricting, given the fragility of *N. meningitidis*), PCR offers the substantial benefit of being able to be performed on dead bacterial cells that have been killed by either refrigeration or previous antimicrobial drug treatment. The simple way of storing and dispatching CSF samples collected in remote areas was convenient and realistic for the circumstances in Niger. Although the biologic confirmation by PCR is retrospective in our study and cannot be used for case management, this surveillance network compensates efficiently for the lack of functional laboratories at the local level outside of the capital. During the 2003 meningitis season, PCR assay allowed satisfactory monitoring of the causative agents of bacterial meningitis and of the involved meningococcal serogroups, which is important in adapting the most appropriate preventive strategy while serogroup W135 has the potential to cause epidemics in the countries of the meningitis belt.

In conclusion, to compensate for the severe shortage of laboratories outside the capital, the PCR assay proved to be a valuable tool for routine microbiologic surveillance of bacterial meningitis in Niger. The country has started implementing the Integrated Disease Surveillance and Response plan within which this microbiologic surveillance is fully integrated.

Ms. Sidikou is a biological engineer and is the head of the molecular biology laboratory in the Centre de Recherches Médicales et Sanitaires (CERMES) in Niamey, Niger. Her major research interest is the enhancement of the microbiologic surveillance of bacterial meningitides.
